# Structural and Practical Departments

**Published:** 1906-08-18

**Authors:** 


### STRUCTURAL AND PRACTICAL DEPARTMENTS.

### URALITE.

Uralite is a material composed in the main of asbestos, and it is so-called because the supply of the material comes largely from the Ural mountains. The process of manufacture is very much the same as that of paper. The raw material is first of all freed from all foreign matter and then mixed in proper proportions and beaten up in a machine with revolving panels into a pulp. The pulp then passes into a machine, which is very much like that used in paper manufacture and is passed through a series of rolls which partially dries it and renders it compact. It then passes to a revolving drum, on which are fixed succeeding layers of the material until the required thickness is obtained, and at this stage a chemical solution is added in order to make the layers adhere together. The sheets as they come from the drum are cut up and then subjected to hydraulic pressure, then they are partially dried and afterwards steeped in a solution of silica, washed, and again dried. The result of all this is to produce a hard, compact material about ⅛in. thick in sheets of 6 feet by 3 feet.

The uses to which it can be put are many. Cut into small sizes, it forms an excellent and very light tile for covering roofs—nailed in large sheets to wooden framing, it can be used for lining both the inside and the outside of enclosures to buildings. Being a non-conductor of heat it forms an excellent lining under roofs. One of its most important qualities is its absolutely fireproof nature—it cannot be burnt, however great the temperature it may be subjected to.

An example of its usefulness in temporary hospitals is the Metropolitan Asylums Board Hospital at Tooting called the "Fountain" Hospital. At this hospital two huts were erected, one lined with Uralite and the other lined with deal match-boarding. They were set on fire, and at the end of half an hour the match-boarded hut was a charred mass, while the hut lined with Uralite had sustained no damage whatever.

For tropical climates we should consider this material particularly suitable both from its fire-resisting quality and for its non-conducting nature. It is not only proof against fire, but against chemical fumes of all sorts. Lastly, it is very light and needs but a very slight framework to support it.

Besides the uses which we have referred to, it is an excellent material for lining fireproof safes. At Earl's Court some few years ago a test was carried out which showed the remarkable fire-resisting character of this material. A small hut was erected, lined inside with Uralite, and in the hut was placed a small box lined inside and out with Uralite and full of papers. The hut was quite full of combustible materials, and these were set alight, and a very high temperature indeed was recorded. When the fire had burnt out and the hut had cooled sufficiently to enter it, the box was removed and opened and the papers were found inside quite intact.

